# The Bereaved Families' Preferences for Individualized Goals of Care for Terminal Dyspnea: What Is an Acceptable Balance between Dyspnea Intensity and Communication Capacity?

**DOI:** 10.1089/pmr.2020.0035

**Published:** 2020-05-14

**Authors:** Masanori Mori, Tatsuya Morita, Kengo Imai, Naosuke Yokomichi, Takashi Yamaguchi, Kento Masukawa, Yoshiyuki Kizawa, Satoru Tsuneto, Yasuo Shima, Mitsunori Miyashita

**Affiliations:** ^1^ Division of Palliative and Supportive Care, Seirei Mikatahara General Hospital, Hamamatsu, Japan.; ^2^ Seirei Hospice, Seirei Mikatahara General Hospital, Hamamatsu, Japan.; ^3^ Division of Palliative Care, Konan Medical Center, Kobe, Japan.; ^4^ Department of Palliative Nursing, Health Sciences, Tohoku University Graduate School of Medicine, Sendai, Japan.; ^5^ Department of Palliative Medicine, Kobe University Graduate School of Medicine, Kobe, Japan.; ^6^ Department of Human Health Sciences, Graduate School of Medicine, Kyoto University, Kyoto, Japan.; ^7^ Department of Palliative Medicine, Tsukuba Medical Center Hospital, Tsukuba, Ibaraki, Japan.

**Keywords:** communication capacity, dyspnea intensity, goals of care, terminal dyspnea

## Abstract

***Background:*** Toward the individualized care of terminally ill patients with dyspnea (“terminal dyspnea”), it is essential to identify individualized goals of care (GOC) to achieve an acceptable balance between dyspnea intensity and communication capacity.

***Objective:*** To explore preferences for individualized GOC for terminal dyspnea, and factors associated with the preferences.

***Design:*** A nationwide cross-sectional survey.

***Setting/Subjects:*** In total, 1055 bereaved families of cancer patients admitted to 167 inpatient hospices in Japan.

***Measurements:*** Preferences for individualized GOC for terminal dyspnea to achieve an acceptable balance between dyspnea intensity and communication capacity, should individuals experience continuous moderate or severe/overwhelming dyspnea despite optimal palliative care, and perceptions about a good death.

***Results:*** Among 548 participants (response rate = 52%), we analyzed responses of 477 families whose loved one suffered dyspnea in the last week of life. In total, 167 (45%; 95% confidence interval [CI] = 40%–50%) and 272 (80%; 95% CI = 75%–84%) participants would prioritize dyspnea relief over communication capacity, should they continuously suffer moderate or severe/overwhelming dyspnea, respectively. In multivariate analyses, the determinants of the prioritization of dyspnea relief were perceiving physical comfort as important for a good death (odds ratio [OR] = 1.389; 95% CI = 1.062–1.818; *p* = 0.017) in moderate dyspnea, and perceiving physical comfort (OR = 2.505; 95% CI = 1.718–3.651; *p* < 0.001) and not perceiving mental awareness (OR = 0.695; 95% CI = 0.529–0.913; *p* = 0.009) as important in severe/overwhelming dyspnea.

***Conclusions:*** Preferences for individualized GOC for terminal dyspnea can vary among individuals and with different symptom intensity, and may be influenced by perceptions about a good death. Outcome measurements incorporating an acceptable balance between dyspnea intensity and communication capacity should be developed.

## Introduction

Dyspnea is one of the most prevalent and distressing symptoms in terminally ill cancer patients, and tends to worsen as death approaches.^1–5^ Effective management of dyspnea in the last weeks to days of life (“terminal dyspnea”) is challenging. Evidence from previous clinical trials conducted in patients with a relatively good condition may not be fully applicable to patients very close to death, and optimal treatment outcomes have not been established.^6,7^ Moreover, terminally ill patients often develop impaired communication capacity associated with the natural course or medications with sedative effects.^6–8^ A recent multicenter observational study involving terminally ill cancer patients with dyspnea revealed that even with the deliberate use of morphine with or without sedatives by palliative care specialists, approximately 30% to 40% of patients continued to experience dyspnea, and >20% became unable to communicate over 48 hours.^6^

Both symptom relief and maintenance of communication capacity are generally considered important for a good death.^9,10^ International experts recently suggested that, toward the individualized care for terminal dyspnea patients, it is vital not only to determine the individualized goal regarding the dyspnea intensity alone,^11,12^ but also to identify individualized goals of care (GOC) to achieve an acceptable balance, or “trade-off,” between dyspnea relief and maintenance of communication capacity.^7,13^ Such preferences may be affected by dyspnea intensity as well as individuals' perceptions about a good death.^9,10^ Currently, however, no established scale is available to measure such bi-dimensional outcomes. Understanding individuals' preferences for GOC for terminal dyspnea may serve as the first step to developing outcomes incorporating an acceptable balance.

To the best of our knowledge, however, no previous studies clarified preferences for individualized GOC for terminal dyspnea to achieve an acceptable balance between dyspnea intensity and communication capacity. Interviewing or administering a questionnaire to patients very close to death who suffer moderate to overwhelming dyspnea despite optimal palliative care is burdensome. Moreover, nonresponse because of impaired communication capacity might result in a biased conclusion.^9^ Bereaved families who have actually cared for their loved ones with terminal dyspnea may provide valuable insight based on their direct observation.

Thus, the primary aim of this study was to explore bereaved families' preferences for individualized GOC for terminal dyspnea to achieve an acceptable balance between dyspnea intensity and communication capacity. We also examined whether their perceptions of a good death contribute to their preferences for individualized GOC.

## Methods

This study was conducted as a part of the Japan Hospice and Palliative Care Evaluation (J-HOPE)-4 study, a cross-sectional anonymous self-reported questionnaire survey.^14^ This was primarily a quality improvement project, regularly performed every three or four years. At this time, of all the 324 inpatient hospices/palliative care units (PCUs) certified by the Hospice Palliative Care Japan, 187 agreed to participate. We asked each institution to identify and consecutively list up to 80 bereaved family members of patients who had died before January 1, 2018; and the total number of potential participants was 14,958 for the entire J-HOPE4 study. The questionnaires comprised two sections: common questionnaires for overall quality measurement and 53 additional questionnaires, one of which was the questionnaire of this study. Additional questionnaires were randomly assigned to the participants in combinations, and 1055 participants from 167 PCUs were included in this study.

### Participants and procedures

A cross-sectional anonymous self-reported questionnaire survey was conducted between May and June 2018. As mentioned earlier, we decided to administer a questionnaire to bereaved family members, as they could provide valuable insight based on direct observation of their loved one with terminal dyspnea. Administering a questionnaire to patients with terminal dyspnea would be burdensome and nonresponse might lead to bias. Administering a questionnaire to the general population would not provide relevant insight, as most members of the general public may not be able to imagine care for terminal dyspnea patients clearly due to the lack of direct experiences. Thus, we included adult bereaved family members of adult patients who died of cancer (one family member for one patient). The exclusion criteria included (1) inability to complete the questionnaire because of health issues such as cognitive impairment or visual disability, (2) bereaved family members of patients with treatment-associated death or death in intensive care units, (3) bereaved family members of patients who had received palliative care services for less than three days (to ensure sufficient length of admission for quality evaluation), and (4) serious psychological distress as determined by the primary physician or a nurse. The final criterion was, as in our previous studies^15–19^ adopted on the assumption that such a physician could identify families who might suffer serious psychological distress due to this study. No formal criteria or psychiatric screening was applied.

Questionnaires were sent to the bereaved family members identified by each participating institution along with an explanation of the survey. Return of the completed questionnaire was considered as indicating consent to participate in the study. We asked participants to return the completed questionnaire to the study secretariat office within one month. We sent a reminder to nonresponders at one month after sending the questionnaire. If they did not wish to participate, they were asked to check a “no participation” box and return the incomplete questionnaire. The ethical and scientific validity of the study was verified by the institutional review board (IRB) at the central institution (Tohoku University, No. 2017-2-236-1; November 20, 2017), followed by IRBs of all participating institutions.

### Questionnaire

The questionnaire first described the context of terminal dyspnea as follows: “We will ask you about breathlessness during the last week of life. During this period, it may be difficult to relieve breathlessness while having communication capacity maintained. In such a case, a doctor is wondering how much communication capacity should be maintained even if a patient suffers breathlessness.” Then, it asked about the patient's dyspnea in the last week of life; family members' preferences for individualized GOC for terminal dyspnea to achieve an acceptable balance between dyspnea intensity and communication capacity; and their perception about a good death. Because of the lack of an existing specific measurement tool to evaluate preferences for individualized GOC for terminal dyspnea patients, we developed the questionnaire for this study based on a systematic literature review, and extensive discussions among the authors.^6,9,10,13,20–23^ Face validity was confirmed by pilot testing and the unanimous agreement of the authors.

#### Family-perceived patients' dyspnea in the last week of life

To identify bereaved family members who experienced care for terminal dyspnea of their loved one, we asked participants if their loved one suffered dyspnea in the last week of life and its overall intensity. Participants responded on a 5-point Likert-type scale (not at all, slightly, moderately, severely, and overwhelmingly) based on the Integrated Palliative care Outcome Scale (IPOS).^23^

#### Preferences for individualized GOC for terminal dyspnea

We asked participants about their preferences for individualized GOC for terminal dyspnea in hypothetical scenarios where they continue to suffer moderate or severe/overwhelming dyspnea in the last week of life despite optimal palliative care. The response options were as follows: “I would wish to be able to communicate fully, even if I continued to suffer dyspnea” (high prioritization of communication capacity), “I would wish to be able to communicate simple matters, even if I continued to suffer dyspnea” (moderate prioritization of communication capacity), and “I would wish to have no dyspnea, even if I could not communicate at all” (high prioritization of dyspnea relief). The first two options were meant to represent prioritization of communication capacity over dyspnea relief, whereas the last option represented prioritization of dyspnea relief over communication capacity. The categories of dyspnea intensity and communication capacity were adopted from IPOS and Communication Capacity Scale.^22,23^

#### Perceptions about a good death

To explore participants' perceptions about a good death, we adopted a conceptual framework based on previous studies on a good death.^9,10,20,21^ We asked participants how important they perceived each of the following three elements to be in the last week of life on a 7-point Likert-type scale from 1 (absolutely unimportant) to 7 (absolutely important): “being free from physical distress,” “being able to say what I wanted to dear people,” and “being mentally aware.”

#### Background data

We also collected background data such as patients' age, gender, and primary cancer site from the participating PCUs, as well as families' age, gender, relationship with the patient, education, and perceived social support from the families. To measure perceived social support from people around them, we utilized the item “degree of supportive listening” derived from the Social Support Scale, a brief, reliable, and widely used scale designed to assess the content of support respondents perceived.^24,25^ The actual question was “how willing are people to listen when you need to talk about your worries or problems?” The participants responded on a 5-point Likert scale (“0: not at all” to “4: a great deal”) with a higher score indicating greater perceived social support.

### Statistical analyses

We used descriptive statistics to summarize the participants' background and calculated the proportion of their responses with a 95% confidence interval (CI). For the purpose of comparisons, respondents to the question regarding the preferences for individualized GOC were divided into two groups: family members who would prioritize communication capacity over dyspnea relief versus those who would prioritize dyspnea relief over communication capacity. This cutoff was determined on the basis of clinical implication as well as the distribution of the actual data to enable division of the entire sample into appropriately sized groups for comparisons.

To explore the potential contributors to participants' preferences for individualized GOC, logistic univariate regression analyses were performed to screen using background characteristics and participants' perceptions about a good death as independent variables, and the participants' preferences for individualized GOC as a dependent variable. Finally, to identify independent determinants of the preferences for individualized GOC, all factors with *p* < 0.1 identified in univariate analyses were entered into multivariate logistic regression analysis. The results of regression analyses are presented as point estimate odds ratios (ORs) with two-sided 95% CIs. In all statistical evaluations, *p*-values of 0.05 or lower were considered significant. Missing data were excluded. All analyses were performed using the Statistical Package for the Social Sciences, version 25.0 (SPSS, Inc., IBM, Japan).

## Results

We sent out 1055 questionnaires, and 656 (63%) were returned. As 108 families refused to participate, there were a total of 548 (52%) responses. Of them, we analyzed responses of 477 (87%) participants who reported that their loved one suffered dyspnea in the last week of life. The mean duration between the day of death and the day when the completed questionnaire was returned was 353 ± 139 days. The baseline characteristics are summarized in Table 1. The mean age of the patients who died of cancer was 76 ± 12 years, and 50% were men. The most frequent primary tumor site was the gastrointestinal tract, followed by the lungs. One hundred seventy-five (37%), 128 (27%), 144 (30%), and 30 (6.3%) patients were reported to have suffered from dyspnea slightly, moderately, severely, and overwhelmingly, respectively, in the last week of life. Participants had a mean age of 63 ± 12 years, and 37% were men. Forty-two percent of the bereaved persons were spouses of the patients.

**Table 1.** Characteristics of Participants (*N* = 477)

Baseline characteristics	Values
Patients	
Age, years (mean ± SD)	76 ± 12
Gender	
Male	238 (50)
Female	239 (50)
Primary cancer sites	
Esophagus, stomach, colon, and rectum	124 (26)
Lung	97 (20)
Liver, gall bladder, and pancreas	91 (19)
Kidney, prostate, and bladder	33 (6.9)
Uterus and ovary	33 (6.9)
Breast	26 (5.5)
Head and neck	19 (4.0)
Blood and lymph nodes (leukemia, lymphoma, and myeloma)	15 (3.1)
Other	39 (8.2)
Family-perceived dyspnea intensity in the last week of life	
Slightly	175 (37)
Moderately	128 (27)
Severely	144 (30)
Overwhelmingly	30 (6.3)
Families	
Age, years (mean ± SD)	63 ± 12
Gender	
Male	175 (37)
Female	298 (63)
Relationship with the patient	
Spouse	201 (42)
Other	274 (58)
Education	
≤High school	271 (57)
University/graduate school	199 (42)
Perceived social support (“degree of supportive listening”)^a^	2.83 ± 0.87
Religion	
Buddhism	264 (55)
Christianity	14 (2.9)
Shintoism	7 (1.5)
Other religion	10 (2.1)
No religion	171 (36)

Values are mean ± SD, or *n* (%). Total percentages do not equal 100% because of missing values.

^a^ Mean of scores of “degree of supportive listening” with responses ranging from “0: not at all” to “4: a great deal” with a higher score indicating greater perceived social support.

SD, standard deviation

### Preferences for individualized GOC

**FIG. 1.**
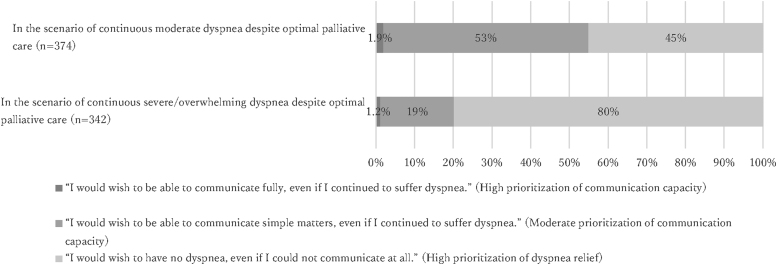
Preferences for individualized goals of care for dyspnea in the last week of life.

In the hypothetical scenario where participants continue to suffer moderate dyspnea in the last week of life despite optimal palliative care, 7 (1.9%; 95% CI = 1%–4%), 200 (53%; 95% CI = 48%–59%), and 167 (45%; 95% CI = 40%–50%) participants answered that “I would wish to be able to communicate fully, even if I continued to suffer dyspnea,” “I would wish to be able to communicate simple matters, even if I continued to suffer dyspnea,” and “I would wish to have no dyspnea, even if I could not communicate at all,” respectively (Fig. 1). In contrast, should they continue to suffer severe/overwhelming dyspnea, 4 (1.2%; 95% CI = 0%–3%), 66 (19%; 95% CI = 15%–24%), and 272 (80%; 95% CI = 75%–84%) participants answered that “I would wish to be able to communicate fully, even if I continued to suffer dyspnea,” “I would wish to be able to communicate simple matters, even if I continued to suffer dyspnea,” and “I would wish to have no dyspnea, even if I could not communicate at all,” respectively.

### Perceptions about a good death

In total, 451 (96%), 392 (84%), and 264 (56%) participants considered “being free from physical distress,” “being able to say what I wanted to dear people,” and “being mentally aware” as important (somewhat important/important/absolutely important), respectively (Table 2).

**Table 2.** Perceptions About a Good Death

	Mean^a^ (SD)	Absolutely unimportant	Unimportant	Somewhat unimportant	Unsure	Somewhat important	Important	Absolutely important
Being free from physical distress (*n* = 471)	6.3 (0.9)	1 (0.2%)	4 (0.8%)	3 (0.6%)	12 (2.5%)	32 (6.8%)	173 (37%)	246 (52%)
Being able to say what I wanted to dear people (*n* = 469)	5.6 (1.2)	0	13 (2.8%)	13 (2.8%)	51 (11%)	108 (23%)	178 (38%)	106 (23%)
Being mentally aware (*n* = 468)	4.9 (1.4)	4 (0.9%)	21 (4.5%)	33 (7.1%)	146 (31%)	92 (20%)	117 (25%)	55 (12%)

^a^ Mean of the responses (1: absolutely unimportant to 7: absolutely important).

### Determinants of the preferences for individualized GOC: Univariate analyses

Univariate analyses revealed that in the scenario where participants continue to suffer moderate dyspnea in the last week of life, those who perceived “being free from physical distress” as important for a good death (OR = 1.294; 95% CI = 1.005–1.666; *p* = 0.046) and those who did not perceive “being able to say what I wanted to dear people” as important (OR = 0.838; 95% CI = 0.705–0.997; *p* = 0.046) were significantly more likely to prioritize dyspnea relief over communication capacity (Table 3). Likewise, in the scenario where participants continue to suffer severe/overwhelming dyspnea, those who perceived “being free from physical distress” as important for a good death (OR = 1.883; 95% CI = 1.381–2.567; *p* < 0.001) and those who did not perceive “being able to say what I wanted to dear people” (OR = 0.779; 95% CI = 0.612–0.990; *p* = 0.042) and “being mentally aware” (OR = 0.686; 95% CI = 0.557–0.846; *p* < 0.001) as important were significantly more likely to prioritize dyspnea relief over communication capacity (Table 3).

**Table 3.** Determinants of the Prioritization of Dyspnea Relief Over Communication Capacity: Univariate Analyses

Variables	In a scenario of continuous moderate dyspnea			In a scenario of continuous severe/overwhelming dyspnea		
	OR	95% CI	*p*	OR	95% CI	*p*
Baseline characteristics						
Patients						
Patient's gender (male [Ref.] vs. female)	0.919	0.611–1.382	0.686	0.819	0.483–1.388	0.458
Patient's age	1.013	0.996–1.030	0.133	1.000	0.978–1.022	0.999
Primary cancer sites						
Esophagus, stomach, colon, and rectum (Ref.)						
Lung	1.084	0.595–1.975	0.792	1.259	0.597–2.657	0.545
Liver, gall bladder, and pancreas	1.112	0.582–2.124	0.748	1.521	0.639–3.622	0.343
Kidney, prostate, and bladder	1.071	0.415–2.766	0.887	1.193	0.351–4.050	0.777
Uterus and ovary	0.918	0.269–3.141	0.892	0.522	0.136–1.998	0.343
Breast	0.918	0.365–2.314	0.857	1.417	0.424–4.735	0.572
Head and neck	0.723	0.286–1.830	0.494	0.767	0.275–2.143	0.613
Blood and lymph nodes (leukemia, lymphoma, and myeloma)	1.800	0.526–6.156	0.349	2.088	0.240–18.178	0.505
Other	1.029	0.466–2.271	0.944	1.253	0.411–3.822	0.692
Family-perceived dyspnea intensity in the last week of life	0.968	0.771–1.216	0.782	0.885	0.679–1.153	0.366
Families						
Family's age	0.995	0.978–1.012	0.589	0.988	0.966–1.011	0.299
Family's gender (male [Ref.] vs. female)	0.752	0.492–1.148	0.187	1.286	0.748–2.210	0.363
Relationship with the patient (spouse [Ref.] vs. other)	1.432	0.937–2.190	0.097	1.197	0.701–2.044	0.509
Education (≤ high school [Ref.] vs. university/graduate school)	0.932	0.616–1.409	0.737	0.961	0.566–1.633	0.883
Perceived social support	0.972	0.769–1.228	0.811	0.783	0.568–1.081	0.138
Religion (no vs. yes)	0.989	0.646–1.513	0.959	0.961	0.559–1.653	0.886
Perceptions about a good death						
Being free from physical distress	1.294	1.005–1.666	0.046	1.883	1.381–2.567	<0.001
Being able to say what I wanted to dear people	0.838	0.705–0.997	0.046	0.779	0.612–0.990	0.042
Being mentally aware	0.867	0.742–1.014	0.075	0.686	0.557–0.846	<0.001

CI, confidence interval; OR, odds ratio; Ref., reference.

### Multivariate analyses

In the scenario where participants continue to suffer moderate dyspnea despite optimal palliative care in the last week of life, the only independent determinant of the prioritization of dyspnea relief over communication capacity was participants perceiving “being free from physical distress” as important for a good death (OR = 1.389; 95% CI = 1.062–1.818; *p* = 0.017) (Table 4). In the scenario where participants continue to suffer severe/overwhelming dyspnea, the independent determinants of the prioritization of dyspnea relief over communication capacity were participants perceiving “being free from physical distress” (OR = 2.505; 95% CI = 1.718–3.651; *p* < 0.001) and those not perceiving “being mentally aware” (OR = 0.695; 95% CI = 0.529–0.913; *p* = 0.009) as important for a good death (Table 4).

**Table 4.** Independent Determinants of the Prioritization of Dyspnea Relief over Communication Capacity: Multivariate Analyses

Variables	In a scenario of continuous moderate dyspnea			In a scenario of continuous severe/overwhelming dyspnea		
	OR	95% CI	p	OR	95% CI	p
Background characteristics						
Relationship with the patient (spouse [Ref.] vs. other)	1.450	0.936–2.244	0.096			
Perceptions about a good death						
Being free from physical distress	1.389	1.062–1.818	0.017	2.505	1.718–3.651	<0.001
Being able to say what I wanted to dear people	0.814	0.653–1.016	0.069	0.716	0.494–1.037	0.077
Being mentally aware	0.968	0.799–1.173	0.742	0.695	0.529–0.913	0.009

Nagelkerke *R*^2^ = 0.047 and 0.175 in scenarios of continuous moderate and severe/overwhelming dyspnea, respectively.

## Discussion

This is, to our knowledge, the first nationwide survey to clarify preferences for individualized GOC for terminal dyspnea, and explore contributing factors. Our findings provide clinically useful insight, as the participating families were those who actually experienced care for their loved ones who suffered dyspnea in the last week of life.

The first and most important finding was that preferences for individualized GOC can vary, and they could change depending on the intensity of dyspnea. In the scenario of continuous moderate dyspnea despite optimal palliative care, the proportion of participants who would prioritize dyspnea relief over communication capacity was less than the proportion of those who would prioritize communication capacity over dyspnea relief. In contrast, in the scenario of continuous severe/overwhelming dyspnea, the proportion of participants who would prioritize dyspnea relief increased markedly. These findings suggest varying preferences among different individuals and situations, and empirically support our previous proposal that outcome measurements incorporating an acceptable balance between the two components be established in the palliation of patients with terminal dyspnea.^7^ Potential strategies may include the development of a composite outcome based on both dyspnea intensity and communication capacity,^6^ and separate measurements of dyspnea and communication capacity.^6,13^ In addition, our finding that a large number of participants would prioritize dyspnea relief indicates that outcome measurements other than patient-reported outcomes are urgently needed to continuously evaluate terminal dyspnea in patients who have lost communication capacity.^7^ As proxy and/or objective measurements were shown to have only a weak correlation with patients' expression of dyspnea, future efforts should be made to develop more valid and reliable measurements.^26^

The second important finding was that the perceptions about a good death, not the baseline characteristics, remained independent factors contributing to the preferences for individualized GOC. Overall, participants who valued physical comfort and those who did not value mental awareness were more likely to prioritize dyspnea relief over communication capacity, when terminal dyspnea should persist despite optimal palliative care. Our results are in line with previous guidelines that stressed the importance of identifying individuals' goals and preferences in the care of dying patients.^27–29^ These suggest that the exploration of their values and in-depth perception may help clinicians promote shared decision making on the individualized GOC and provide goal-concordant care.^9,10,20,21,30,31^

Despite the strengths of the nationwide survey, our study has several limitations. First, this was an after-death survey among bereaved family members with a moderate response rate (52%), and the population evaluated was heterogeneous in many ways. There were variable lengths between the patient's death and survey administration; families' emotions and recall might vary from the time of death, and their recall of the emotions and preferences for GOC might change over time as grief is resolved. In addition, there were some missing data. All of these may have introduced recall and selection biases. Previous national surveys involving bereaved family members also reported similar response rates.^10,32^ Second, although we developed outcomes to explore individualized GOC based on the previous studies, we performed no formal testing of the validity and reliability. However, established tools to measure individualized GOC for terminal dyspnea patients were not available, and our findings were highly interpretable. Third, due to the nature of the cross-sectional study, we could not control for the actual treatment for dyspnea in the last week of life. Families might have various experiences of different care approaches for terminal dyspnea. Thus, future prospective studies should develop validated measurements for terminal dyspnea incorporating an acceptable balance, which would help improve treatment strategies to provide individualized care.

In conclusion, this nationwide survey revealed that preferences for individualized GOC for terminal dyspnea patients to achieve an acceptable balance between dyspnea intensity and communication capacity varied widely, and perceptions about a good death influenced the preferences. Future efforts should be made to develop outcome measurements incorporating an acceptable balance between dyspnea intensity and communication capacity, which would help improve individualized care for patients with terminal dyspnea.

## Abbreviations Used

CI: confidence interval
GOC: goals of care
IRB: institutional review board
IPOS: Integrated Palliative care Outcome Scale
J-HOPE: Japan Hospice and Palliative Care Evaluation
OR: odds ratio
PCUs: palliative care units
SD: standard deviation
